# Stress Erythropoiesis is a Key Inflammatory Response

**DOI:** 10.3390/cells9030634

**Published:** 2020-03-06

**Authors:** Robert F. Paulson, Baiye Ruan, Siyang Hao, Yuanting Chen

**Affiliations:** 1 Department of Veterinary and Biomedical Sciences, Center for Molecular Immunology and Infectious Disease at Penn State University, University Park, PA 16802, USA; 2 Department of Veterinary and Biomedical Sciences, Pathobiology Graduate Program at Penn State University, University Park, PA 16802, USA; bur27@psu.edu; 3 Department of Veterinary and Biomedical Sciences, Graduate Program in Molecular, Cellular and Integrative Biosciences at Penn State University, University Park, PA 16802, USA; sxh442@psu.edu (S.H.); yxc257@psu.edu (Y.C.)

**Keywords:** stress erythropoiesis, erythroblastic islands, pro-inflammatory cytokines, erythrophagocytosis, anemia of inflammation

## Abstract

Bone marrow medullary erythropoiesis is primarily homeostatic. It produces new erythrocytes at a constant rate, which is balanced by the turnover of senescent erythrocytes by macrophages in the spleen. Despite the enormous capacity of the bone marrow to produce erythrocytes, there are times when it is unable to keep pace with erythroid demand. At these times stress erythropoiesis predominates. Stress erythropoiesis generates a large bolus of new erythrocytes to maintain homeostasis until steady state erythropoiesis can resume. In this review, we outline the mechanistic differences between stress erythropoiesis and steady state erythropoiesis and show that their responses to inflammation are complementary. We propose a new hypothesis that stress erythropoiesis is induced by inflammation and plays a key role in maintaining erythroid homeostasis during inflammatory responses.

## 1. Bone Marrow Erythropoiesis Maintains Homeostasis at Steady State

Erythropoiesis in an adult is a constant process with an enormous capacity to generate erythrocytes. It has been estimated that adult humans make more than 2.5 × 10^6^ erythrocytes per second, whereas adult mice make approximately 7000 erythrocytes per second [1,2]. Despite this high level of production, steady state erythropoiesis is primarily homeostatic. The rate of production is offset by the turnover of senescent erythrocytes by red pulp macrophages in the spleen [3]. This balance between production and destruction is tightly regulated to maintain an optimal erythrocyte concentration in the blood. Too few erythrocytes are unable to provide sufficient oxygen delivery to the tissues, whereas too many erythrocytes lead to high blood viscosity, impairing blood flow, which also decreases oxygen delivery.

Although a comprehensive review of the mechanisms that regulate erythrocyte production is beyond the scope of this review, a broad discussion of how steady state erythropoiesis fits into hematopoiesis will provide a foundation for understanding the relationship between stress erythropoiesis and steady state erythropoiesis. Functional assays first defined the committed erythroid progenitors referred to as BFU-E and CFU-E [4]. Flow cytometry and transcriptome analysis, which has progressed from microarray analysis to single cell RNAseq (scRNAseq), have greatly refined these progenitor populations (for examples, see [5,6,7,8,9,10]). We now understand that multipotential progenitors (MPPs) are primarily responsible for homeostatic hematopoiesis [11,12]. MPPs develop along a trajectory that generates lineage committed progenitors (for examples, see [12,13,14]). If we focus on the erythroid lineage, many cell surface markers have been identified that mark developmental transitions during erythropoiesis [5,6,7,10,15]. However, erythroid development is really a continuum of developmental states that define the path from MPP to erythrocyte [5,6,7,8,10]. In addition, hematopoiesis generates other myeloid and lymphoid lineages each having their own trajectory of development and requirements for homeostasis [13,14,16]. The problem for hematopoiesis is how to integrate the requirement for erythroid homeostasis with the production of other myeloid and lymphoid lineages. Chronic stimulation of erythropoiesis with Epo significantly changes the composition of bone marrow progenitor populations. Lymphoid-primed MPPs and myeloid committed progenitors like granulocyte-macrophage progenitors, GMPs, and megakaryocyte progenitors all decrease when erythroid progenitors increase. Transcriptomics shows that Epo alters the gene expression programs of hematopoietic stem cells, HSCs, to drive erythropoiesis [17,18]. The ability of Epo to skew hematopoiesis towards erythropoiesis is illustrated by the work of Tusi et al. using scRNAseq analysis of Kit+ hematopoietic progenitor cells, which provides a framework to understand the impact of skewing hematopoiesis to one lineage [8]. They showed that under conditions of erythroid stimulation (treatment with Epo) erythroid production increased, but the increase in erythroid-biased and erythroid progenitors was offset by decreases in progenitors for other lineages. This observation suggests a model of hematopoiesis that defines a developmental continuum from MPP to terminally differentiated hematopoietic cells. Because this continuum is finite, an increased demand for myeloid cells in response to infection or inflammation leads to a decrease in erythroid production. This model is supported by multiple observations. The KRN model of inflammatory arthritis leads to a myeloid skewing of bone marrow hematopoiesis characterized by increased numbers of GMPs and peripheral CD11b+ cells, which is offset by decreased numbers of megakaryocyte-erythroid progenitors, MEPs, which results in anemia [19]. Similarly, mutation of microRNA-146a, which negatively regulates NFκβ signaling, results in increased pro-inflammatory cytokine expression, which causes myeloid skewing, inhibition of lymphoid development and anemia that is compensated by splenic erythropoiesis [20,21,22]. Chronic treatment of mice with IL-1β leads to increased myeloid cell production, decreased lymphoid cell production and anemia [23,24]. These data show that bone marrow hematopoiesis responds to inflammatory signals by increasing myeloid cell production but reducing lymphoid and erythroid output. In addition, other mechanisms such as increased turnover of cells in the periphery or loss of mature cells through apoptosis can exacerbate this decrease. This loss of mature cells is especially problematic in the erythroid lineage where any prolonged drop in erythroid output could compromise oxygen delivery to tissues and lead to anemia. In order to compensate for this decrease in erythropoiesis, an extramedullary source of erythropoiesis that operates outside the developmental continuum of the bone marrow must be induced, and that source is stress erythropoiesis.

## 2. Stress Erythropoiesis Maintains Erythroid Homeostasis When Steady State Erythropoiesis is Impaired

For more than fifty years, analysis of anemic stress in mice showed that they compensated for low bone marrow erythroid output by increasing splenic erythropoiesis [25,26,27,28,29]. Anemic mice exhibited splenomegaly associated with increased iron uptake, which was incorporated into hemoglobin, and increased numbers of erythroblasts. Early work on this response to anemia took advantage of phenylhydrazine (PHZ) injection to induce acute hemolytic anemia and analyzed the response in the bone marrow and spleen. This work showed that PHZ injection led to a decrease in BFU-E and multilineage CFU-Mix in the bone marrow, which was compensated by increased BFU-E, CFU-E and CFU-Mix in the spleen [25,26,30,31]. Progenitors were also identified in the peripheral blood. This led to a model where erythropoietic stimulation mobilized progenitors to migrate from the bone marrow to the spleen where they finished differentiation [25]. This model is based on the hypothesis that the erythroid progenitors that respond at steady state in the bone marrow are the same as the erythroid progenitors in the spleen that respond to anemia. However, the analysis of the murine *flexed-tail (f)* mutant suggested a different model. These mice exhibit a fetal neonatal anemia that resolves approximately 2 weeks after birth. After which they exhibit normal steady state erythropoiesis with normal numbers of BFU-E and CFU-E in the bone marrow [32,33,34,35]. However, the *f*/*f* mice exhibit a delayed recovery with a more pronounced anemia when challenged with PHZ-induced acute anemia [27,36]. These data suggested that the *f* locus regulated the erythroid response to anemia but did not regulate steady state erythropoiesis. This idea was supported by analysis of the phenotype in *f/f* mice showing that the inability to respond to anemic stress correlated with a defect in the expansion of endogenous erythroid progenitors in the spleen [27,34]. These data suggested a new model where endogenous splenic stress erythroid progenitors used in stress erythropoiesis were distinct from steady state erythroid progenitors [29].

The cloning of the *f* locus in 2005 showed that *f* encoded the transcription factor *Smad5* [27,37]. This discovery changed the model of stress erythropoiesis. Smad5 is phosphorylated and activated by the receptors for bone morphogenetic proteins (BMPs), a family of growth factors that previously had not been associated with erythropoiesis. BMP4 was identified as the key signal in the spleen [27,38,39,40]. The response of BFU-E to BMP4 distinguishes splenic BFU-E from bone marrow BFU-E. Furthermore, splenic BFU-E exhibited different growth properties. Unlike bone marrow BFU-E, which require Epo and a second factor to form colonies, splenic BFU-E only require Epo [27]. This new class of progenitors were termed stress BFU-E and further characterization of these new progenitors showed that in addition to BMP4 and Epo, Stem Cell Factor (SCF) and hypoxia provided the minimum set of factors needed to recapitulate, in vitro, the expansion of stress BFU-E observed in vivo during the recovery from PHZ-induced anemia [38].

**Figure 1:**
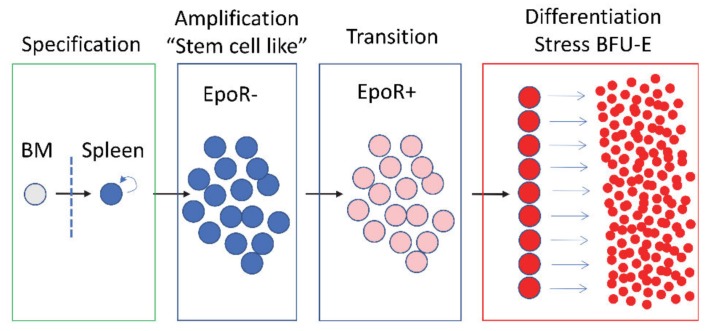
Schematic of stress erythropoiesis. Stress erythropoiesis proceeds through four stages. BM—bone marrow, EpoR—erythropoietin receptor, BFU-E—Burst forming units erythroid.

These initial observations demonstrated that stress erythropoiesis uses signals and progenitor cells that are distinct from steady state erythropoiesis. Further analysis using in vivo models such as erythroid short-term radioprotection-following bone marrow transplant and sterile inflammation models combined with analysis using in vitro stress erythropoiesis cultures expanded the model for stress erythropoiesis [40,41,42]. The in vitro culture system also demonstrated that human stress erythroid progenitors (SEPs) required the same signals as murine SEPs and mutations that affect murine SEP development also affect human SEP development [40,43]. This model separates stress erythropoiesis into four stages, which provides a foundation for understanding the strategy of stress erythropoiesis (Figure 1). Unlike steady state erythropoiesis, which constantly produces erythrocytes, stress erythropoiesis generates a bolus of new erythrocytes derived from the synchronous differentiation of progenitor cells. The initial stage of stress erythropoiesis is the specification of the stress erythroid fate [40,42]. Bone marrow short-term reconstituting hematopoietic stem cells (ST-HSCs–CD34+Kit+Sca1+Lin^neg^) migrate to the spleen where Hedgehog (HH) ligands act in concert with BMP4 to specify the stress erythroid fate. Conditional mutation of the HH receptor *Smoothened (Smo)* or blocking BMP4 signaling with Noggin inhibits the development of stress erythroid progenitors (SEPs) in the spleen. Furthermore, conditional deletion of *Patched (Ptc),* which leads to constitutive HH signaling in the bone marrow, results in the development of BMP4 responsive stress BFU-E in the bone marrow. These data show that the compartmentalization of HH signaling to the spleen is what promotes the extramedullary nature of stress erythropoiesis [39,42].

The next stage of development is the expansion of a transient amplifying population of immature stress progenitors. SEPs proliferate at a rapid rate during this stage. During bone marrow transplant, donor SEPs contribute to 80% of the spleen cells and the spleens of recipient animals become 2–3 fold larger [40]. In vivo and in vitro analysis showed that the proliferating SEPs are made up of three distinct populations. All three populations can be serial transplanted, but are erythroid restricted [40]. Transcriptomics analysis showed that the most immature of these populations express a number of pattern recognition receptors present on myeloid cells and other genes involved in self-renewal of stem cells. Furthermore, these proliferating cells express genes and metabolites involved in anabolic processes needed to generate building blocks for cell division, which support their rapid proliferation [44]. Several signals promote the expansion of these early progenitor cells. GDF15, a TGFβ family member, potentiates the hypoxia response by repressing VHL, the E3 ubiquitin ligase that promotes the degradation of the hypoxia inducible transcription factor Hif1α. GDF15-dependent signaling increases glucose metabolism in proliferating SEPs by increasing the Hif1α-dependent transcription of glycolytic enzymes [44].

In addition to GDF15, corticosteroids are well known to play a role in regulating the expansion of SEPs. Mutation of the glucocorticoid receptor in mice compromises the expansion of progenitor cells in the spleen [45]. The role of glucocorticoids in regulating stress erythropoiesis was further revealed by the analysis of fetal liver erythropoiesis, which shares many characteristics with stress erythropoiesis as mutations that affect stress erythropoiesis also have defects in fetal liver erythropoiesis [46,47]. Synthetic glucocorticoids like dexamethasone promote the proliferation of fetal liver erythroid progenitors in vitro [48,49,50,51,52,53,54]. The glucocorticoid receptor synergizes with Hif1α to drive the proliferation of immature erythroid progenitors [7,55]. These factors maintain the expression of genes that inhibit differentiation and slow the induction of the erythroid differentiation program [54]. Their combined effect in vivo allows for the expansion of a population of immature SEPs that do not differentiate until the numbers of progenitors are high enough to generate sufficient erythrocytes to maintain homeostasis. This property of SEPs is best illustrated using the bone marrow transplant assay. Immature SEPs that rapidly proliferate in the spleen following bone marrow transplant (BMT) are erythroid restricted but are not committed to differentiation. They fail to generate stress BFU-E colonies until day 8 after transplant [42]. This time point corresponds to the increase in Epo mRNA expression in the kidney and the increase in serum Epo levels. In vitro analysis confirmed that Epo promoted the transition to differentiation, and its effect is potentiated by hypoxia [40]. Thus, Epo signaling plays a central role in converting this transient amplifying population of SEPs into stress BFU-E committed to differentiation. It also provides a means to regulate the erythroid output during the response to anemic stress. How the timing of Epo expression in the kidney is regulated in relation to the proliferation of erythroid progenitors is not understood. However, the timing of this signal is crucial as it impacts the size of the pool of immature progenitors. Early induction of Epo would result in insufficient progenitors whereas later induction of Epo would generate more immature progenitors that differentiate into erythrocytes. Coordination of proliferation and differentiation is important for maintaining balance between erythrocyte number and blood viscosity, which optimizes oxygen delivery.

Having a signal that drives the switch from proliferation to differentiation also promotes a more synchronous development of new erythrocytes. The overall process of terminal differentiation of SEPs is similar to steady state progenitors. However, there are differences that distinguish stress erythropoiesis. Unlike steady state progenitors, which down regulate Kit expression and, increase CD71 and Ter119 expression, SEPs maintain expression of Kit receptors even during late stage differentiation [38]. In humans, fetal erythropoiesis is characterized by the expression of gamma globin instead of the adult beta globin. This expression leads to the production of fetal hemoglobin, HbF. In adults the gamma globin gene is repressed, but most humans express low levels of HbF, approximately 1–5% in circulating erythrocytes (for recent reviews, see [56,57]). This percentage increases in patients with inherited bone marrow failure syndromes and during recovery from bone marrow transplant [58,59,60]. Unfortunately, in vivo analysis of human stress erythropoiesis focuses on studying patients with congenital anemias or following bone marrow transplant, which limits the scope of the experiments. To circumvent this problem, we developed human stress erythropoiesis cultures using the same culture conditions that expand a population of murine SEPs. The human SEPs generated in these cultures express the same cell surface markers, with the exception of Sca1, and have the same growth factor requirements as mouse SEPs. Analysis of human SEPs expanded in vitro showed that they expressed high levels of gamma globin relative to adult beta globin, and the erythrocytes generated in vitro expressed HbF [40]. BCL11A, which represses the expression of gamma globin in adults [61,62,63] is not expressed in in vitro generated human SEPs. This loss of BCL11A is consistent with the increased gamma globin expression. Murine SEPs show a similar change in globin gene expression. In this case the lack of Bcl11a expression leads to increased expression of the mouse embryonic βh1 globin [40]. These data demonstrate that humans and mice generate similar populations of SEPs and these progenitors exhibit properties like fetal erythroid progenitors.

## 3. Inflammation Inhibits Steady State Erythropoiesis

Stress erythropoiesis was initially analyzed in the context of acute anemia induced experimentally by PHZ injection or bone marrow transplant. Although these experimental systems robustly induce stress erythropoiesis, in the clinic these anemias would be treated by blood transfusion, which inhibits stress erythropoiesis. Furthermore, acute blood loss is such a rare event that it is unlikely to have contributed to the evolution of stress erythropoiesis response. In contrast, infection and inflammation are common events that shape the evolution of physiological responses. Infection and inflammation induce the anemia of inflammation (AI), which, after iron deficiency anemia, is the most common anemia observed in hospitalized patients. AI is caused by inflammatory responses that impact three areas of erythrocyte production and homeostasis [64,65,66,67,68]. Inflammation leads to iron sequestration that limits iron availability for hemoglobin synthesis [68,69,70]. Pro-inflammatory cytokines alter bone marrow hematopoiesis to increase the production of innate immune effector cells and inhibit steady state erythropoiesis [24,67,71,72,73,74,75,76]. These signals also increase erythrocyte turnover in the periphery [71]. These responses embody the double-edged sword that is inherent to the immune response to infection and tissue damage.

From an immune system perspective, sequestering iron from pathogens prevents this essential micronutrient from promoting pathogen proliferation. The decrease in iron availability results in part from increased IL-6 production, which induces hepcidin production in the liver [77,78]. Hepcidin acts as a hormone and binds to the primary cellular iron exporter, ferroportin (Fpn), inducing its degradation. In addition, other pro-inflammatory cytokines as well as lipopolysaccharides from bacteria can directly inhibit Fpn expression leading to defects in iron export [79,80,81,82,83,84]. This loss of Fpn effectively sequesters iron from pathogens, limiting their growth. However, from the perspective of erythropoiesis, sequestering iron limits iron availability for hemoglobin production.

Another effect of pro-inflammatory cytokines is the skewing of hematopoietic production to increase myeloid effector cells that are needed to fight infection. This change in hematopoiesis comes at two levels, the active promotion of myelopoiesis and the inhibition of bone marrow erythropoiesis. Inflammation increases the production of IL-1β, which promotes the production of myeloid progenitors and inhibits erythroid development. IL-1β acts on MPPs by increasing the expression of *Pu.1*, which in turn promotes myeloid development and inhibits the erythroid program [23,24,85]. Similarly, interferon gamma (Ifnγ) signaling through the induction of *Irf1* also activates the expression of *Pu.1* in MPPs leading to myeloid skewing of hematopoiesis [71,86]. The shift from erythropoiesis to myelopoiesis is also directly promoted by the activation of Toll-like receptors (TLRs) on ST-HSCs and MPPs by pathogen specific ligands. Activation of MyD88 dependent signaling downstream of these receptors activates NFκβ and significantly increases the production of pro-inflammatory cytokines [87]. This increase in cytokine expression and in particular the expression of IL-6 drives the production of innate effector cells. Furthermore, myeloid cells can be directly produced from MPPs rather than developing through intermediates like granulocyte macrophage progenitors [88,89,90].

To accommodate this increase in myelopoiesis, pro-inflammatory factors such as tumor necrosis factor alpha (TNFα) actively inhibit erythropoiesis through direct and indirect mechanisms. TNFα signaling inhibits Gata1 dependent gene expression, leading to decreased erythroid differentiation and apoptosis of erythroid progenitors [67,72,75,76]. TNFα signaling also indirectly regulates erythropoiesis by regulating Epo mRNA expression in the kidney [85,91]. The hypoxia inducible transcription factor Hif1α drives the expression of Epo, and TNFα signaling blocks Hif1α activity through the action of NFκβ and Gata2. These examples as well as others demonstrate that the increased production of innate immune effector cells in response to infection and inflammation comes with a cost as steady state production of erythrocytes is inhibited.

The increase in myeloid effector cells also leads to changes in erythrocyte half-life. Senescent erythrocytes are routinely removed from circulation by erythrophagocytosis in the spleen, but pro-inflammatory cytokines and, in particular, Ifnγ increase erythrocyte turnover [3,71,92]. Increased hemophagocytosis plays an important role in the clearance of dead and infected cells during the resolution of infection. However, phagocytosis of erythrocytes, platelets and leukocytes during infection can lead to cytopenias and anemia. Akilesh et al. showed that TLR7 and TLR9 signaling induces the development of hemophagocytes that are derived from Ly6C^hi^ monocytes [93]. Expansion of this population of splenic hemophagocytes during *Plasmodium* infection leads to the turnover of bystander uninfected erythrocytes and development of malarial anemia [94,95].

## 4. Inflammation Induces Stress Erythropoiesis

Infection and inflammation from tissue damage are common occurrences, but the decrease in steady state erythropoiesis in healthy individuals is compensated so that a potentially lethal anemia is not observed. Studies in mice demonstrated that splenic erythropoiesis increases in experimental models of infection and sterile inflammation [41,96,97,98]. Mice infected with *Salmonella* develop anemia, but it is compensated by increased erythropoiesis in the spleen. Inflammatory signaling through TLRs plays a key role in this response as mice with mutations in the downstream signaling molecules, Myd88 and TRIF, fail to induce splenic erythropoiesis after infection. *Salmonella* infection also increases Epo production by the kidney and liver, which contributes to the increase in splenic stress erythropoiesis [99,100,101]. Like the infection models, sterile inflammation models show increased splenic erythropoiesis. Mice injected with heat killed *Brucella abortus* develop anemia over a 2-week period. This anemia is associated with decreased bone marrow erythropoiesis but, by day 7 after treatment, splenic erythropoiesis increases and the mice recover by 4 weeks [96,97,102]. Similarly, mice treated with the TLR2 ligand, zymosan A, develop a generalized inflammatory disease that is characterized by anemia that is resolved by 4 weeks after treatment. The recovery from zymosan A induced anemia is associated with increased splenic stress erythropoiesis [41,98]. These sterile inflammation models show all the hallmarks of AI, increased hepcidin expression and iron restriction, decreased steady state erythropoiesis coupled with increased myeloid output and increased erythrocyte turnover. All these conditions are detrimental to steady state erythropoiesis, but stress erythropoiesis compensates for these effects of inflammation and maintains homeostasis.

The ability of mice in these model systems to recover from anemia suggests that stress erythropoiesis responds differently to inflammation and can function when steady state erythropoiesis is impaired. Analysis of mice treated with zymosan A showed that stress erythropoiesis is induced within 24 h of treatment, which occurs before the mice develop anemia [41]. This observation ran counter to the prevailing models of stress erythropoiesis, which relied on hypoxia as a key inducer of stress erythropoiesis because BMP4 and Epo are hypoxia inducible genes. The ability of inflammation to rapidly induce stress erythropoiesis suggested that other mechanisms activate stress erythropoiesis in response to inflammation. After treatment with zymosan, phagocytosis of erythrocytes increases in the spleen because TLR2 signaling decreases macrophage expression of Sirpα, a receptor that normally inhibits phagocytosis [41,92]. Breakdown of hemoglobin by these macrophages releases heme, which in turn leads to the heme-dependent increase in the expression of the transcription factor SpiC [103]. GDF15 is a critical signal for the initiation of stress erythropoiesis and its expression is regulated by SpiC. Although it can be pathological, in the context of stress erythropoiesis, inflammation induced erythrophagocytosis, acts as an initiating signal. Increased GDF15 signaling in the spleen boosts hypoxia dependent BMP4 expression by stabilizing Hif1α through the repression of VHL [44]. These signals promote the expansion of the SEP population in the spleen. They are aided by pro-inflammatory cytokines. Unlike bone marrow, steady state erythropoiesis, which is inhibited by these cytokines TNFα and, to a lesser extent, IL-1β promote the proliferation of SEPs [41]. As the SEPs proliferate, increased expression of Epo by the kidney promotes the transition of SEPs to stress BFU-E. Epo also induces the expression of erythroferrone (Erfe), which antagonizes hepcidin and releases iron for the differentiation of stress BFU-E [104,105,106]. These events lead to a rapid induction of stress erythropoiesis where stress BFU-E are generated over the next 6 days and the percentage of reticulocytes steadily increases during the 14-day recovery period [41,98]. These data show that response to inflammatory signals actively promotes stress erythropoiesis, which compensates for the loss of steady state erythropoiesis.

The response of splenic macrophages and monocytes to inflammatory signals underscores their key role in regulating inflammation-induced stress erythropoiesis. In addition to GDF15 and BMP4, erythrophagocytosis also leads to increased expression of pro-inflammatory cytokines and chemokines. These signals drive the recruitment of monocytes into the spleen [28]. Unlike the bone marrow, where erythroblastic islands (EBIs) are maintained for steady state erythropoiesis, the splenic stress erythropoiesis niche expands in concert with the SEPs. The majority of the EBIs in the spleen contain monocyte derived macrophages. These monocytes mature during recovery. As SEPs are differentiating, monocytes also differentiate along a developmental continuum from monocyte to pre-red pulp macrophage to red pulp macrophage. The most immature SEPs (CD34+CD133+Kit+Sca1+) interact with monocytes whereas mature stress BFU-E (Kit+Sca1^neg^CD71+TER119+) interact with pre-red pulp macrophages and red pulp macrophages [28]. These observations highlight a new question—what signals regulate the co-development of the SEPs and the niche? As mentioned above, glucocorticoids affect the proliferation and differentiation of SEPs. They have also been shown the affect the development of monocytes and macrophages and regulate their interaction with erythroid progenitors. CD14+ monocytes from humans improve erythroid differentiation in CD34+ hematopoietic cultures [107]. Treatment with glucocorticoids promotes the differentiation of these monocytes into macrophages that express CD169 and CD163, and other markers that are associated with EBI macrophages [108]. Glucocorticoids also increase the motility of the macrophages and promote their interactions with erythroblasts [109]. These data show that glucocorticoids represent a signal that could coordinate SEP and niche development.

Although Epo plays a key role in regulating erythroid development, emerging data suggest that Epo signaling in macrophages regulates erythropoiesis [110]. Work from Li et al. showed that EBI macrophages express the Epo receptor (EpoR) [111]. Although the precise role for macrophage Epo receptor signaling in erythroid development has not been identified, EpoR signaling macrophages is known to inhibit inflammatory responses and changes the expression of cytokines produced by macrophages [112]. A potential role for macrophages in regulating erythropoiesis is illustrated in myeloproliferative disease. Deregulated JAK2 signaling down stream of EpoR drives the expansion and differentiation of erythroid cells in Polycythemia vera. However, expression of the mutant JAK2^V617F^ allele in myeloid cells also leads to polycythemia and myeloproliferative disease suggesting that deregulated EpoR signaling macrophages profoundly affects the erythroid development [113]. These observations dovetail with earlier work showing that depletion of macrophages blocks the pathology in a murine Polcythemia vera model [114,115]. The role of aberrant macrophage signaling during erythropoiesis is not limited to over production of erythrocytes. Macrophages also play role in promoting the inefficient erythropoiesis observed in thalassemia. Depletion of macrophages using clodronate liposomes rescued the anemia of thalassemia mice (Hbb^th3/+^) [115]. Studies will be needed to further understand the impact of inflammatory signals on the niche and how the interplay between inflammatory signals the signals produced by macrophages and monocytes impact the generation of new erythrocytes by stress erythropoiesis.

## 5. New Model: Stress Erythropoiesis is a Key Component of the Inflammatory Response

Inflammation due to infection or tissue damage is a common occurrence during one’s lifetime. The immune system rapidly responds to these conditions by mobilizing innate effector cells, skewing steady state hematopoietic production from erythropoiesis to myelopoiesis, sequestering iron and other nutrients from pathogens and increasing erythrophagocytosis. All these events are driven by pro-inflammatory cytokines and play a key role in the immune response. Unfortunately, these responses come with a cost as bone marrow steady state erythropoiesis is inhibited. Data from our lab and others suggest that the loss of steady state erythropoiesis is compensated by extra-medullary stress erythropoiesis [41,96,97]. We observe that inflammatory signals that inhibit bone marrow steady state erythropoiesis support stress erythropoiesis, which generates erythrocytes until the source of the inflammation is neutralized and hematopoiesis in the bone marrow can resume steady state production.

Using this model as a guide for further investigation, several outstanding questions remain. The data from several labs show that the niche and macrophages play key roles in regulating erythropoiesis in the bone marrow and in extramedullary sites, but the response of erythroid progenitors to pro-inflammatory cytokines is site specific. Some of these differences will be due to intrinsic differences between SEPs and steady state erythroid progenitors, but other aspects of the response will be governed by differences in the microenvironment. During stress erythropoiesis, macrophages express GDF15 and BMP4 [28,41], which promote the proliferation and differentiation of SEPs. Similarly, increased levels of Tnfα and IL-1β increase SEP proliferation in vitro, whereas Ifnγ plays a role in recruiting monocytes to the expanding splenic stress erythropoiesis niche [28,41]. The question going forward is whether other pro-inflammatory cytokines function to promote stress erythropoiesis and how pro-inflammatory signals are integrated with other known stress erythropoiesis signals. How do these signals change in pathological conditions like thalassemia and myeloproliferative disease? At present most of the data address how the inflammation impacts erythropoiesis, but an equally intriguing question is whether there is a reciprocal interaction where extra-medullary stress erythropoiesis feeds back to help resolve inflammation. At a systemic level, the rise in serum Epo levels drives the transition from proliferating SEPs to differentiating progenitors, but Epo is also known to inhibit inflammation by decreasing NFκβ signaling [112]. Thus, it is possible that induction of Epo expression contributes to the resolution of inflammation. Similarly, increased levels of corticosteroids play an essential role in stress erythropoiesis, but glucocorticoid receptor signaling is also a potent anti-inflammatory signal [116]. Thus, Epo and corticosteroids represent signals that drive the proliferation and differentiation of SEPs and promote the resolution of inflammation. The dual role of these signals is consistent with a model suggesting that inflammation induces stress erythropoiesis to compensate for the decrease in steady state erythropoiesis, but also as part of an overall mechanism to resolve inflammation and return to steady state hematopoiesis. Persistent inflammation is pathological and leads to AI. Acute inflammation induces stress erythropoiesis, but the strategy of stress erythropoiesis is the periodic production of waves of new erythrocytes. In chronic inflammatory disease, it is likely that if chronic inflammation is present, periodic waves of stress erythropoiesis will occur. Our data show that this period in mice is approximately 28 days. SEP proliferation and differentiation takes 7 days after which it takes 21 days to replenish progenitors and the niche in the spleen [39]. We observed the same period in mice treated with zymosan, which exhibited a cyclic anemia with a period of approximately 28 days [41]. Therefore, in AI, persistent inflammation could induce cyclical episodes of anemia and recovery, which overtime could lead to a breakdown in the stress erythroid response. Understanding how chronic inflammation affects this process and how it overwhelms the stress erythropoiesis system will be the key to developing new therapies to treat AI.

In conclusion, stress erythropoiesis shares many signals and responses with inflammation, which suggest that it functions as a normal component of the inflammatory response to infection and tissue damage.
